# Bayesian Regulators to Promote Sharp Image Edges in Limited-Angle Tomography by Combining $L_{1}$ and $L_{2}$ norms

**Published:** 2026-03-21

**Authors:** Gengsheng L Zeng

**Affiliations:** 1 Department of Computer Science, Utah Valley University, Orem, USA.; 2 Department of Radiology and Imaging Sciences, University of Utah, Salt Lake City, USA.

**Keywords:** Bayesian objective function, Total variation, Edge preservation, L_0_, L_1_, L_2_, L_q_ optimization

## Abstract

When the image reconstruction problem is severely ill-conditioned, for example, when the scanning angle is small, the analytic reconstruction algorithms produce images with too many artifacts to be useful in practice, while iterative Bayesian reconstruction algorithms can produce better images. The main purpose of Bayesian constraints is to regulate and stabilize the algorithm. Bayesian penalty minimization is a popular and effective method in regulating, denoising, and edge preserving. The main players for the Bayesian regulators are the total-variation (TV), Huber, $L_{0},L_{1},L_{2}$, and $L_{q}$ norms. This paper suggests some other methods to regulate an iterative algorithm and to encourage sharp image edges. Some computer simulations are provided. The $L_{q}$-like regulators seem to be more effective than Huber regulators in terms of edge-preserving. In this paper, we propose the combination of the user-friendly $L_{1}$ and $L_{2}$ norms to approximate the user-unfriendly $L_{q}$ norm.

## Introduction

In incomplete-data tomography, for example, in limited angle tomography, the image reconstruction problem is severely ill-conditioned [1,2]. Without regularization, the reconstructed images contain too many artifacts to have any practical applications. An effective way to incorporate regularization is through an iterative algorithm that has Bayesian terms. The total variation (TV) regulator is effective in stabilizing the reconstruction procedure, reducing noise, preserving sharp edges, and promoting piecewise-constant images.

In section II, two versions of the famous TV functionals for two-dimensional images are discussed. These two versions are isotropic TV and anisotropic TV [3]. Huber functional is another popular functional used to denoise and preserve the sharp image edges [4]. Some other *ad hoc* Huber-lookalike functionals are suggested in this paper. The suggested new Huber-lookalike functionals are shown to be more effective in sharp-edge preserving than the original Huber functional.

In section III, some limited-angle computer simulations are carried out. The Huber-lookalike functions are compared when they are used in an iterative algorithm.

We believe that deep-learning-based image reconstruction is another form of Bayesian image reconstruction, which is not in the scope of this paper.

## Methods

### Isotropic TV and Anisotropic TV Regulators

Anisotropic TV of a two-dimensional (2D) image F(x,y) is defined as

(1) $$TV_{\text{aniso}}(F)=\iint \|\nabla F{\|}_{1}\text{dxdy}$$

with the local $L_{1}$ norm of the image gradient magnitude as

(2) $$\|\nabla F{\|}_{1}=\left|\nabla_{x}F\right|+\left|\nabla_{y}F\right|.$$

At every point (x,y), the gradient of a 2D function F(x,y) is a vector $\left(\frac{\partial F}{\partial x},\frac{\partial F}{\partial y}\right)$. The $L_{1}$ norm this gradient vector is defined as $\left|\frac{\partial F}{\partial x}\right|+\left|\frac{\partial F}{\partial y}\right|$. Therefore, the anisotropic TV of image F(x,y) is the summation of the $L_{1}$ norms the gradient vectors $\left(\frac{\partial F}{\partial x},\frac{\partial F}{\partial y}\right)$ over all points (x,y).

If we replace the $L_{1}$ norm with the local $L_{2}$ norm at each point (x,y), $\left|\frac{\partial F}{\partial x}\right|+\left|\frac{\partial F}{\partial y}\right|$ becomes $\sqrt{{\left(\frac{\partial F}{\partial x}\right)}^{2}+{\left(\frac{\partial F}{\partial y}\right)}^{2}}$. As a result, the isotropic TV of a 2D image F(x,y) is defined as the summation of the local $L_{2}$ norms the gradient vectors $\left(\frac{\partial F}{\partial x},\frac{\partial F}{\partial y}\right)$ over all points (x,y):

(3) $$TV_{iso}(F)=\iint \|\nabla F{\|}_{2}\text{dxdy}.$$

with the local $L_{2}$ norm of the image gradient magnitude as

(4) $$\|\nabla F{\|}_{2}=\sqrt{{\left(\nabla_{x}F\right)}^{2}+{\left(\nabla_{y}F\right)}^{2}.}$$

We point out that the integral in (3) is the sum of the square-roots, which are the “local” $L_{2}$ norms of the partial gradients at a point. On the other hand, the $L_{2}$ norm of the gradient images is the square-root of the sum of the squared partial gradient images with respect to x and with respect to y, as expressed in (5) below:

(5) $$\|\nabla F{\|}_{2}=\sqrt{\iint \left[{\left(\nabla_{x}F\right)}^{2}+{\left(\nabla_{y}F\right)}^{2}\right]\text{dxdy}}.$$

The name ‘isotropic TV’ implies that the value of $TV_{\text{iso}}(F)$ is rotational invariant. Let us consider a rotated coordinate system

(6) St=cosθsinθ−sinθcosθxy.

Then we have

(7) $${\left(\frac{\partial F}{\partial s}\right)}^{2}+{\left(\frac{\partial F}{\partial t}\right)}^{2}={\left(\frac{\partial F}{\partial x}\text{cos}\theta +\frac{\partial F}{\partial y}\text{sin}\theta \right)}^{2}+{\left(-\frac{\partial F}{\partial x}\text{sin}\theta +\frac{\partial F}{\partial y}\text{cos}\theta \right)}^{2}\;={\left(\frac{\partial F}{\partial x}\right)}^{2}{\text{cos}}^{2}\theta +2\frac{\partial F}{\partial x}\frac{\partial F}{\partial y}\text{cos}\theta \text{sin}\theta +{\left(\frac{\partial F}{\partial y}\right)}^{2}{\text{sin}}^{2}\theta +{\left(\frac{\partial F}{\partial x}\right)}^{2}{\text{sin}}^{2}\theta -2\frac{\partial F}{\partial x}\frac{\partial F}{\partial y}\text{cos}\theta \text{sin}\theta +{\left(\frac{\partial F}{\partial y}\right)}^{2}{\text{cos}}^{2}\theta \;={\left(\frac{\partial F}{\partial x}\right)}^{2}+{\left(\frac{\partial F}{\partial y}\right)}^{2}.$$

The anisotropic TV is sensitive to horizontal/vertical edges, while the isotropic TV is rotation invariant.

### Huber Regulators

A Huber regulator is expressed as a combination of a quadratic penalty for small values and a linear penalty for large values as follows.


(8) $$H(g)=\left\{\begin{array}{cc} \frac{1}{2}g^{2}, & |g|\leq c\\ c|g|-\frac{1}{2}c^{2}, & |g|>c. \end{array}\right.$$

In (8), g is image gradient $\sqrt{{\left(\nabla_{x}F\right)}^{2}+{\left(\nabla_{y}F\right)}^{2}}$ given in (4).

### Huber-Lookalike Regulators

We now define some more regulators. Similar to the Huber regulator, we will define some regulators that have different expressions in different regions. We set a threshold c. When the magnitude of the gradient is greater than c,|g|>c, the gradient of the regulator is assigned as a zero-mean random variable. When |g|<c, three regulators are defined as

(9) $$I(g)=\frac{1}{2}g^{2};$$

(10) $$J(g)=\|\nabla F{\|}_{2}-\frac{\alpha }{2}g^{2};$$

(11) $$K(g)=\|\nabla F{\|}_{1}-\frac{\alpha }{2}g^{2}.$$

In the region of |g|<c, regulator I in (9) is the square of the local $L_{2}$ norm of the image gradient, that is, ${\left(\nabla_{x}F\right)}^{2}+{\left(\nabla_{y}F\right)}^{2}$.

In the region of |g|<c, regulator J in (10) is a combination of $TV_{\text{iso}}(F)$ and the $L_{2}$ norm. The parameter a can be positive or negative.

In the region of |g|<𝒞, regulator J in (11) is a combination of $TV_{\text{aniso}}(F)$ and the $L_{2}$ norm. To make sure J and K are nondecreasing, we require that 1−αc≥0, that is, α≤1/c.

**Figure 1:**
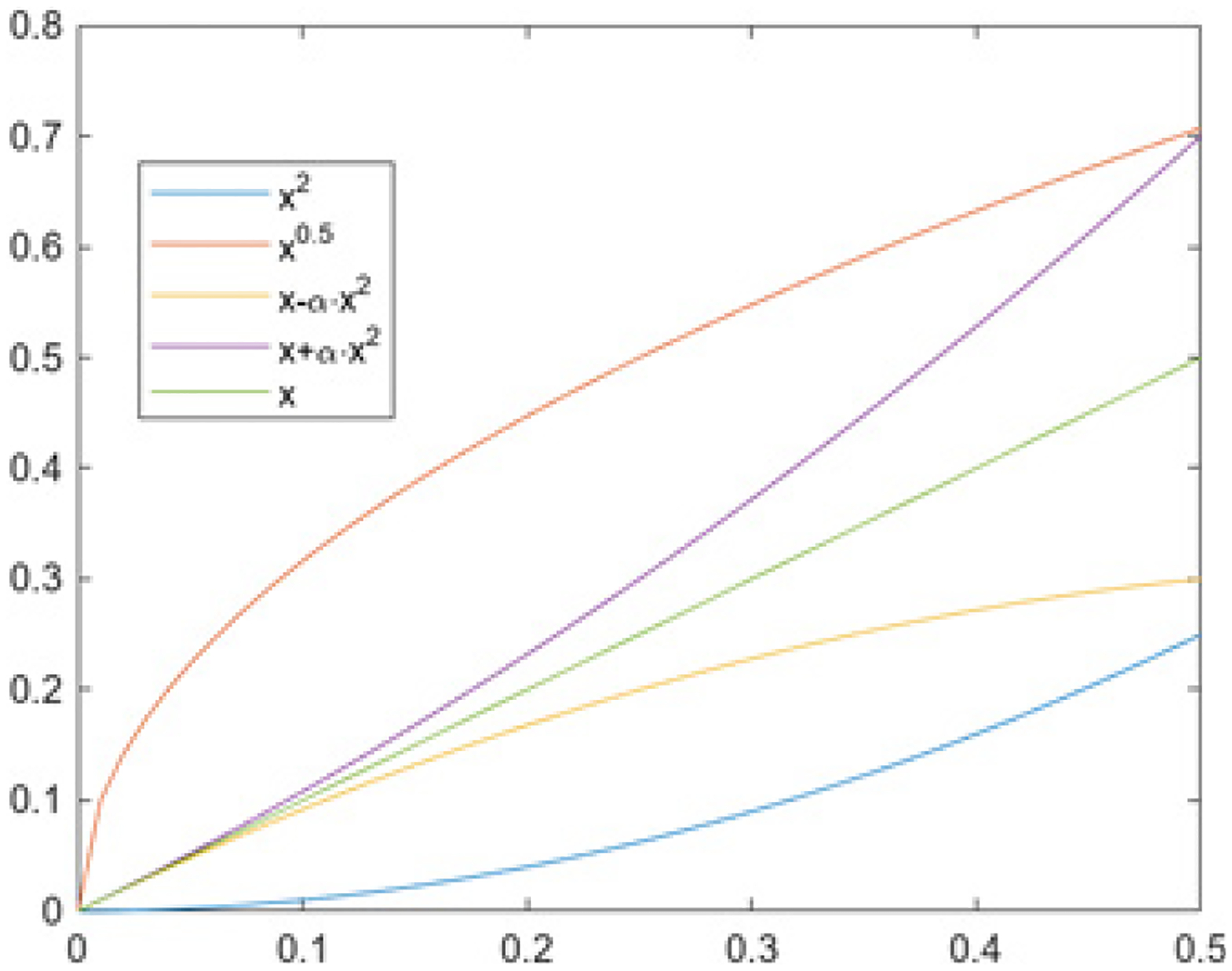
Some monotonic functions for 0≤x≤0.5.

Both local $L_{1}$ norm and local $L_{2}$ norm behave like an $L_{1}$ norm. The motivation for these combinations is to approximate an $L_{q}$ norm, with 0<q<1. Some curves are shown on the interval [0, 0.5] in Figure 1. If the second order derivative of the function is positive (negative), the curve is convex (concave). The $L_{q}$ norm is defined by the function of $y=|x{|}^{q}$ [5]. If 0<q<1 and $x>0,y^{\prime \prime }=q(q-1)x^{\left(q-2\right)}<0$. Thus, $y=x^{q}$ is concave. The function $y=x-\alpha x^{2}$ for x>0 and α>0 is also concave. An advantage of using $y=x-\alpha x^{2}$ to replace $y=x^{q}$ is that x=0 is a singular point for the first order derivative of $y=x^{q}$. On the other hand, there is no singularity for any order derivative of $y=x-\alpha x^{2}$.

**Figure 2:**
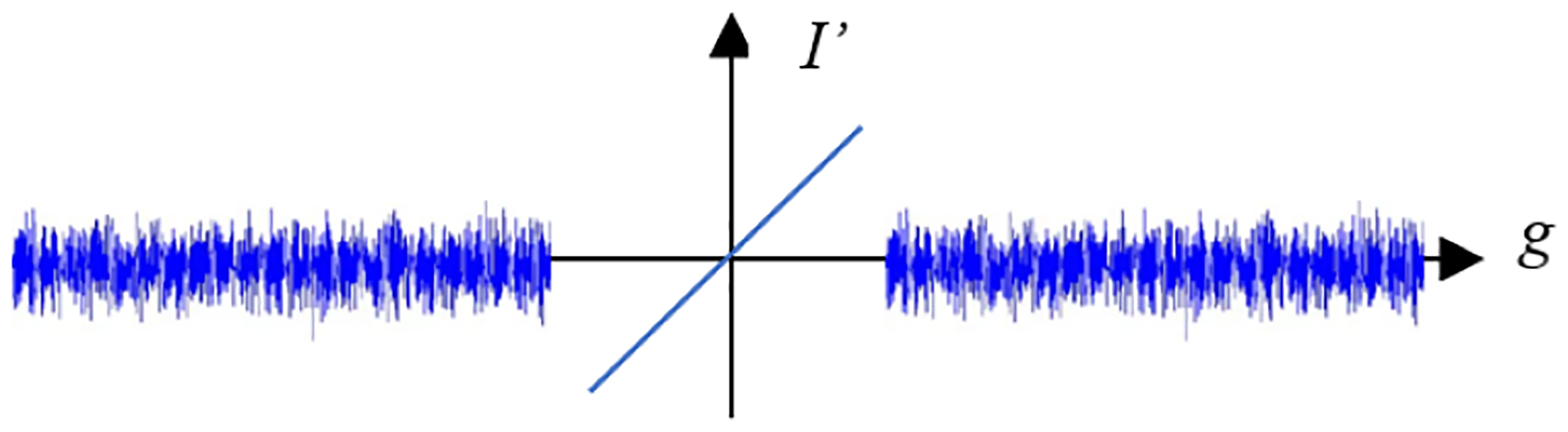
The gradient of penalty I.

**Figure 3:**
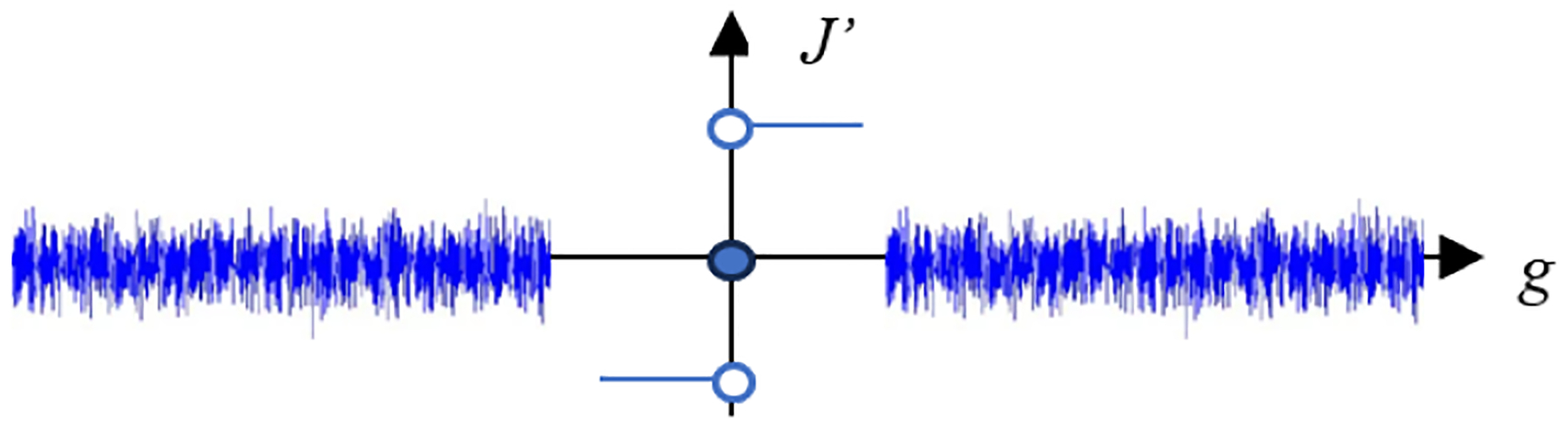
The gradient of penalty J with α=0.

**Figure 4:**
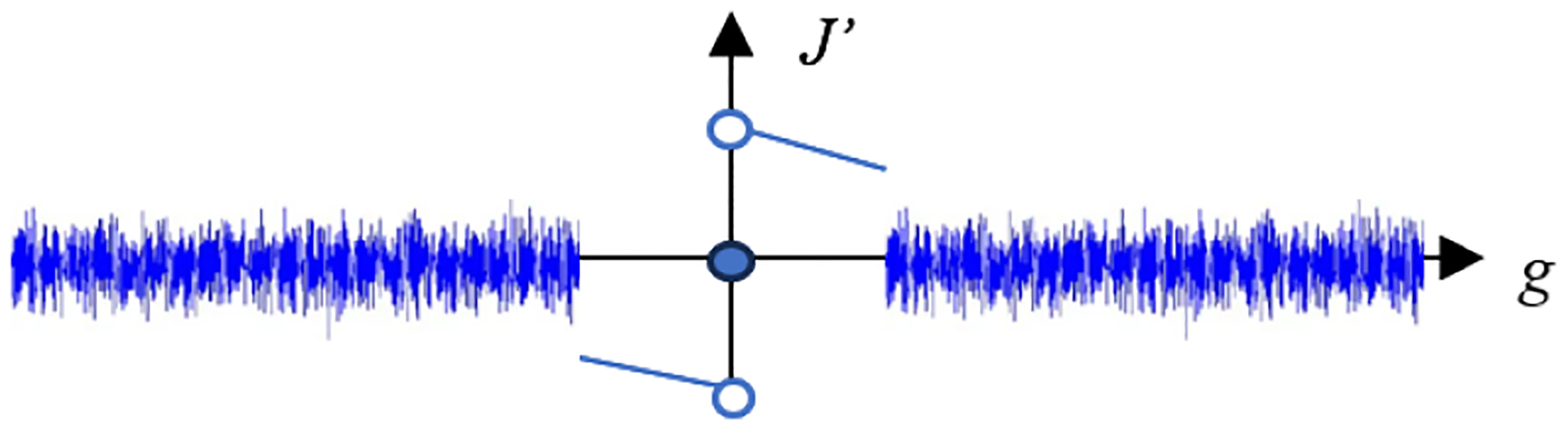
The gradient of penalty J with α>0.

**Figure 5:**
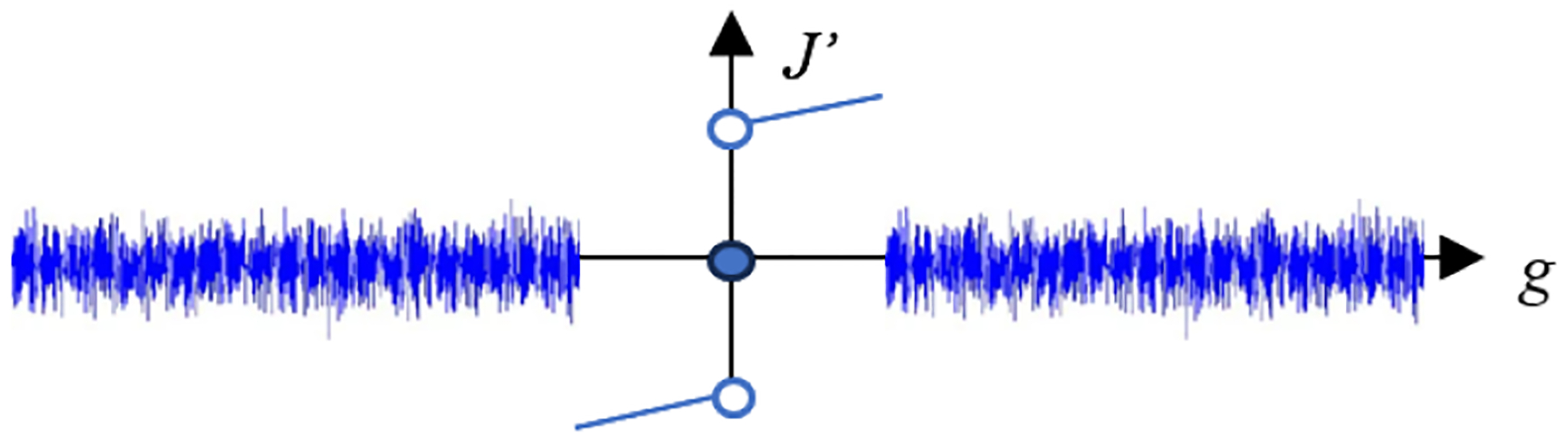
The gradient of penalty J with α<0.

Some one-dimensional profiles of derivative cases are depicted in Figures 2–5, respectively. The purpose of ‘zero mean random’ is to give the algorithm a chance to get out of a local minimum during an optimization procedure.

### Computer Simulations

A computer simulated noiseless phantom, and its rotated version were used in the computer simulations. All images were 256×256. The imaging geometry was parallel-beam, and the scan-angle was 40° with 400 views.

There are many image reconstruction algorithms. In this paper, we adopted a “Projections onto Convex Sets” (POCS) algorithm [6], in which the images were reconstructed with 10000 iterations, each iteration containing 10 iterations of the “Maximum-Likelihood Expectation-Maximization” (MLEM) algorithm [7] and one iteration of the gradient descent algorithm [8]. The MLEM algorithm enforces data fidelity, and the gradient descent algorithm minimizes the regulation function of the image gradient.

When the magnitude of the gradient is less than parameter c, the Regulator I (9) and Huber Regulator (8) are the $L_{2}$ norm of the gradient. The parameter c was set as 0.5 in our simulations. The phantom took 4 values: 0, 0.5, 1, and 1.5. The parameter α was set as 0, 0.001, and −0.001, respectively.

## Results

**Figure 6:**
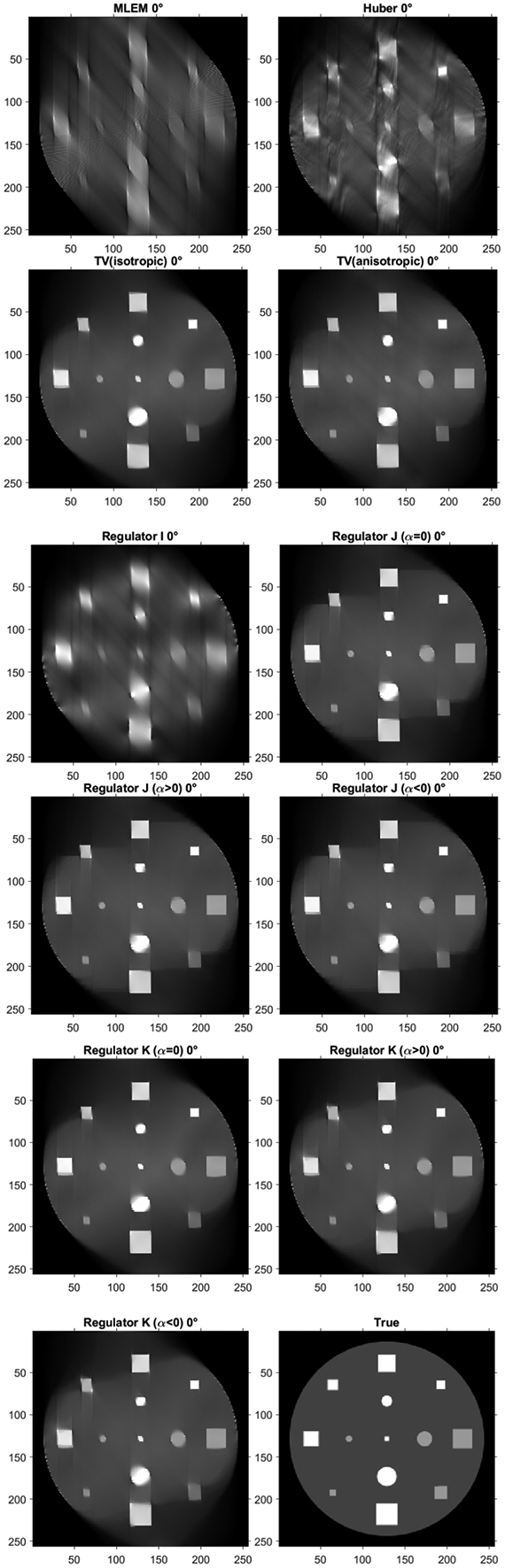
Reconstructed images without phantom rotation.

**Figure 7:**
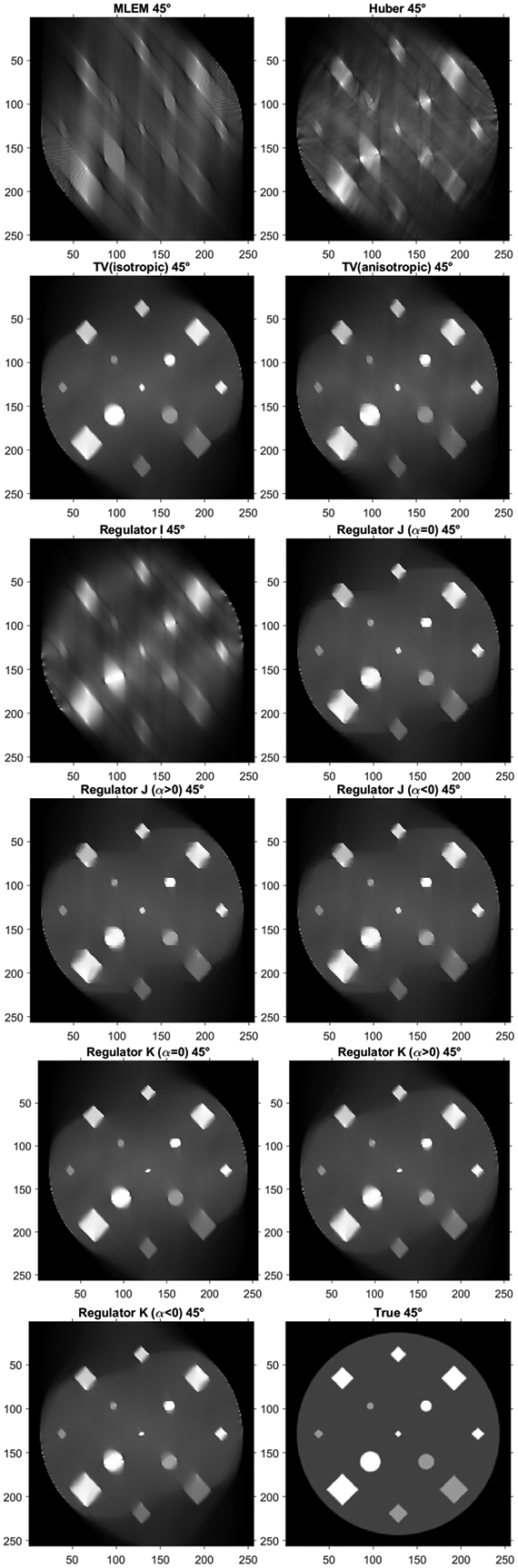
Reconstructed images with phantom rotation by 45°.

Two sets of reconstructed images are shown in this section. The first set of the reconstructed images are shown in Figure 6. After rotating the phantom by 45°, the second set of the reconstructed images are shown in Figure 7. The structural similarity index measure (SSIM) metrics [9] are calculated and listed in Table 1 for comparisons.

The scanning angle was 40°, which is too small to provide enough data for any useful reconstructions. The purpose of the comparison studies here is to illustrate the different effectiveness of the regulators.

## Discussions and Conclusions

Bayesian regulators are effective in image reconstruction especially when projection data is incomplete, for example, in limited angle tomography. Popular Bayesian regulators include the TV norms, $L_{1}$ norm, $L_{0}$ norm, and $L_{q}$ norm. Taking the derivative of an $L_{0}$ norm or an $L_{q}$ norm can be problematic, due to the singularity at the origin when 0≤q<1.

This paper proposes the use of regulator $y=|x|-\alpha /2|x{|}^{2}$ for a small positive α to replace the $L_{q}$ regulator $y=|x{|}^{q}$ for 0<q<1. When x>0, both functions are positive (if 0<α≤2/c); the first-order derivative functions are positive (if 0<α≤1/c); the second-order derivative functions are negative (if 0<α). When x→0, the first-order derivative function of $y=|x|-\alpha /2|x{|}^{2}$ has finite left and right limits; however, the first-order derivative function of $y=|x{|}^{q}$ does not have finite left and right limits.

Our computer simulation examples demonstrate better performance of our proposed regulators than the popular TV regulators and the Huber regulator.

## Figures and Tables

**Table 1:** SSIM for reconstructions (A bigger value is a better value).

Regulator	0° (Figure 6)	45° (Figure 7)
MLEM	0.5417	0.5420
Huber	0.5540	0.5828
TV(isotropic)	0.7451	0.7447
TV(anisotropic)	0.7417	0.7297
I	0.6285	0.6191
J,α=0	0.7455	0.7383
J,α=0.01	0.7450	0.7379
J,α=−0.01	0.7457	0.7378
K,α=0	0.7461	0.7443
K,α=0.01	**0.7498**	**0.7517**
K,α=−0.01	**0.7498**	0.7515
